# Near-Complete Genome Sequences of 12 Coxsackievirus Group A Strains from Hand, Foot, and Mouth Disease and Herpangina Cases with Different Clinical Symptoms

**DOI:** 10.1128/MRA.01655-18

**Published:** 2019-02-21

**Authors:** Shao-Jian Xu, Ya-Qing He, Ren-Li Zhang, Hong Yang, Xiang-Jie Yao, Hai-Long Zhang, Jun Meng, Long Chen, Qi-Hui Lin

**Affiliations:** a District Key Laboratory for Infectious Disease Prevention and Control, Longhua Center for Disease Control and Prevention, Shenzhen, China; b Major Infectious Disease Control Key Laboratory and Shenzhen Public Service Platform of Pathogenic Microorganisms Repository, Institute of Pathogen Biology, Shenzhen Center for Disease Control and Prevention, Shenzhen, China; KU Leuven

## Abstract

Coxsackievirus group A (CV-A) strains are important pathogens of hand, foot, and mouth disease and herpangina. We report here the near-complete genome sequences of 12 CV-A strains isolated from infants and children with different clinical diseases. The presented data will be very useful for future genome-based epidemiological studies.

## Abstract (precis)

Coxsackievirus group A (CV-A) strains are important pathogens of hand, foot, and mouth disease and herpangina. We report here the near-complete genome sequences of 12 CV-A strains isolated from infants and children with different clinical diseases.

## ANNOUNCEMENT

Coxsackievirus group A (CV-A) strains belong to the genus *Enterovirus* in the family *Picornaviridae* (http://www.picornaviridae.com/enterovirus/enterovirus.htm). Enteroviruses (EVs) are a group of naked positive single-stranded RNA viruses, and their genome comprises a 5′ untranslated region (UTR), four capsid proteins (VP1 to VP4), seven nonstructural proteins (2A to 2C and 3A to 3D), and a 3′ UTR (1). EVs are associated with various human diseases, such as hand, foot, and mouth disease (HFMD), herpangina (HA), encephalitis, myelitis, upper and lower respiratory diseases, conjunctivitis, and gastroenteritis (2). Coxsackievirus A2 (CV-A2), CV-A6, CV-A10, and CV-A16 strains were frequently associated with HFMD and HA outbreaks and sporadic infections worldwide over the years (3–7).

In recent years, emerging CV-A2, CV-A6, and CV-A10 strains were frequently detected in HFMD and HA patients in Shenzhen, China. In order to investigate the genetic characteristics of these strains, a total of 12 CV-A strains associated with different clinical diseases, collected between 2012 and 2015, were selected for amplification of the genome sequences. Detailed epidemiological data for these strains (listed in Table 1) demonstrate that CV-A strains display considerable phenotypic variation. Viral RNA was extracted directly from fecal samples or anal swab specimens using a High Pure viral RNA kit (Roche). These strains were typed by real-time reverse transcription (RT)-PCR and seminested RT-PCR (8). Then, a pair of universal primers, EVA-F30 (5′-TTAAAACAGCCTGTGGGTTGTACCCACCCA-3′) and EVA-R36 (5′-GCTATTCTGGTTATAACAAATTTACCCCCACCAGTC-3′), targeting the 5′ UTR and 3′ UTR of the enterovirus A strains, respectively, were used to amplify near-complete genomes of these strains by a one-step RT-PCR method, as described previously (9). The genome sequence of each strain was amplified independently, and a DNA product of ∼7,400 bp was determined using a primer-walking method (TaKaRa, Dalian, China) (10). Contigs of ∼800 bp sequenced by the Sanger method were assembled into a genome sequence using the program Sequencher version 4.9.

**TABLE 1:** Epidemiological data and genome sequence information for 12 coxsackievirus group A strains from this study

Isolate	Sex	Age (mo)	Clinical manifestations	GenBank accession no.	Length in nt (% GC content)^*a*^	Closest strain (% nt identity)
CVA2/Shenzhen50/CHN/2012	Male	36	Fever, herpangina, lethargy, tachypnea	KX595281	7,400 (48.9)	CVA2/Shenzhen133/CHN/2013 (98.8)
CVA2/Shenzhen133/CHN/2013	Female	10	Fever, vesicles on mouth, herpangina	KX595282	7,400 (48.8)	CVA2/Shenzhen50/CHN/2012 (98.8)
CVA2/Shenzhen143/CHN/2013	Male	8	Fever, vesicles on mouth, herpangina, coughing, rhinorrhea, pharyngalgia	KX595283	7,400 (48.8)	431306 (98.7)
CVA2/Shenzhen21/CHN/2015	Male	15	Fever, rash, vesicles on hand, foot, and mouth, myoclonic jerk	KX595284	7,312 (49.1)	BJ13-53/BJ/CHN/2013 (97.7)
CVA6/Shenzhen87/CHN/2014	Male	9	Vesicles on hand, mouth, elbow, and knees, pharyngalgia	KX595285	7,434 (47.0)	SHAPHC5298/SH/CHN/14 (98.7)
CVA6/Shenzhen94/CHN/2014	Male	13	Fever, rash, vesicles on hand, foot, mouth, elbow, knees, and buttock	KX595286	7,434 (47.1)	Hyogo9205 (97.2)
CVA6/sHFMD14/Shenzhen/2015	Male	27	Fever, rash, vesicles on hand, foot, mouth, elbow, knees, and buttock, pharyngalgia, herpangina, tachypnea, aseptic encephalitis	MH716144	7,434 (47.4)	Weifang/SD/CHN/2014 (98.0)
CVA10/Shenzhen152/CHN/2013	Male	4	Fever, rash, vesicles on hand, foot, and mouth	KX595287	7,411 (47.7)	FY05/AH/CHN/2013 (97.0)
CVA10/Shenzhen18/CHN/2014	Male	16	Fever, rash, vesicles on hand, foot, and mouth, pharyngalgia, limb shaking	KX595288	7,411 (47.5)	CV-A10/P911/2013/China (99.1)
CVA10/Shenzhen180/CHN/2014	Female	8	Fever, vesicles on mouth, herpangina	KX595289	7,411 (47.9)	FY05/AH/CHN/2013 (97.7)
CVA10/Shenzhen10/CHN/2015	Female	13	Fever, rash, vesicles on hand, foot, mouth, and buttock, myoclonic jerk	KX595290	7,411 (47.6)	CV-A10/P670/2013/China (98.1)
CVA16/Shenzhen179/CHN/2014	Female	24	Fever, rash, vesicles on hand and foot	KX595295	7,409 (47.4)	YN10-02 (97.9)

a nt, nucleotide.

The genome length, GC content, and most closely related strain in GenBank of the 12 CV-A strains of this study are summarized in Table 1. The four CV-A2 strains of this study show 1.2% to 3.6% nucleotide (nt) differences across the entire genome and 0.6% to 1.1% amino acid (aa) differences across the entire polyprotein. A neighbor-joining phylogenetic tree constructed by using the program MEGA7.0.26 (11), based on the complete VP1 gene of the four CV-A2 strains of this study and CV-A2 reference strains retrieved from GenBank, indicated that the four CV-A2 strains of the study clustered to genogroup D according to the genotyping method proposed by Yang et al. (12). The three CV-A6 strains from this study show 3.5% to 7.4% nt differences and 0.9% to 1.7% aa differences from each other. Phylogenetic analysis based on the complete VP1 sequence showed that these three CV-A6 strains belong to subgenogroup D3 (13). The four CV-A10 strains of the study show 3.7% to 4.8% nt differences and 0.9% to 1.4% aa differences from each other. These four CV-A10 strains belong to subgenogroup C2 based on phylogenetic analysis of the VP1 gene (14). The CV-A16 strain CVA16/Shenzhen179/CHN/2014 was assigned to subgenotype B1b based on phylogenetic analysis of the VP1 gene (15).

### Data availability.

The near-complete genome sequences of 12 coxsackievirus group A strains from the present study have been deposited in DDBJ/ENA/GenBank under the accession numbers listed in Table 1.
